# Osteocytic cells exposed to titanium particles increase sclerostin expression and inhibit osteoblastic cell differentiation mostly via direct cell‐to‐cell contact

**DOI:** 10.1111/jcmm.17460

**Published:** 2022-06-28

**Authors:** Hao Chai, Zai Hang Zhang, Jing Yi Fang, Chang She, De Chun Geng, Wei Xu

**Affiliations:** ^1^ Department of Orthopedics The Second Affiliated Hospital of Soochow University Suzhou Jiangsu Province China; ^2^ The Experiment Center The Medical College of Soochow University Suzhou Jiangsu Province China; ^3^ Department of Orthopedics The First Affiliated Hospital of Soochow University Suzhou Jiangsu Province China

**Keywords:** osteoblast, osteocyte, osteolysis, SOST/sclerostin, wear debris

## Abstract

The mechanism underlying induction of periprosthetic osteolysis by wear particles remains unclear. In this study, cultured MLO‐Y4 osteocytic cells were exposed to different concentrations of titanium (Ti) particles. The results showed that Ti particles increased expression of the osteocytic marker SOST/sclerostin in a dose‐dependent manner, accelerated apoptosis of MLO‐Y4 cells, increased the expression of IL‐6, TNF‐α and connexin 43. SOST silence alleviated the increase of MLO‐Y4 cells apoptosis, decreased the expression of IL‐6, TNF‐α and connexin 43 caused by Ti particles. The different co‐culture systems of MLO‐Y4 cells with MC3T3‐E1 osteoblastic cells were further used to observe the effects of osteocytic cells' changes induced by Ti particles on osteoblastic cells. MLO‐Y4 cells treated with Ti particles inhibited dramatically differentiation of MC3T3‐E1 cells mostly through direct cell‐to‐cell contact. SOST silence attenuated the inhibition effects of Ti‐induced MLO‐Y4 on MC3T3‐E1 osteoblastic differentiation, which ALP level and mineralization of MC3T3‐E1 cells increased and the expression of ALP, OCN and Runx2 increased compared to the Ti‐treated group. Taken together, Ti particles had negative effects on MLO‐Y4 cells and the impact of Ti particles on osteocytic cells was extensive, which may further inhibit osteoblastic differentiation mostly through intercellular contact directly. SOST/sclerostin plays an important role in the process of mutual cell interaction. These findings may help to understand the effect of osteocytes in wear particle‐induced osteolysis.

## 1 INTRODUCTION

It is well known that joint arthroplasty can relieve pain and enhance life quality for patients with severe osteoarthritis or other joint diseases. However, with the significant increase in the number of joint replacements, the problem of aseptic loosening of the prosthesis is becoming more serious. Prosthetic loosening is the main reason for implant failure and the leading cause of revision arthroplasty. Kurtz et al.^1^ projected that the revisions of hip and knee replacement would grow 137% and 601% separately from 2005 to 2030. Periprosthetic osteolysis, in which wear particles induce a biological reaction in histocytes, is the most important factor in aseptic loosening. Wear debris from prosthetic implant grind can activate many types of cells surrounding the prosthesis, including macrophages, monocytes, fibroblasts and osteoclasts,^2^, ^3^, ^4^ which release TNF‐α, IL‐6, MMP‐2 and other pro‐inflammatory cytokines. These pro‐inflammatory cytokines further stimulate osteoclast differentiation and maturation, leading to bone resorption.^5^, ^6^, ^7^ Meanwhile, these pro‐inflammatory cytokines also suppress osteoblastic bone formation and further stimulate the expression of mediators, which participate in the communication between osteoblasts and osteoclasts such as OPG and RANKL, increasing ratio of RANKL/OPG further promoting bone resorption.^8^, ^9^, ^10^ These cells changes disturb the delicate balance between bone formation and bone resorption and cause to periprosthetic osteolysis eventually, which is an unbalanced remodelling in favour of resorption. However, the precise mechanism of periprosthetic osteolysis still remains unclear.

Osteocytes are terminally differentiated osteoblasts, which are deeply buried in the bone matrix. Osteocytes are the major type of bone cells and comprise 90% of all bone cells. Some studies indicate that osteocytes take part in controlling bone formation and bone resorption. Osteocyte released immunostimulatory molecules to the bone surface, promoting the production of proinflammatory cytokines, including TNF‐α, IL‐6 and IL‐1, which inhibited osteoblast differentiation and maturation.^11^ Osteocyte can promote osteoblastic bone formation through canonical Wnt signal pathway.^12^, ^13^ Apoptosis of osteocytes promotes osteoclastic bone resorption, with increased osteoclast number and activity.^14^, ^15^ In particular, the production of SOST/sclerostin in bone exclusively by osteocytes has gained attention because of its role in regulating bone formation and bone resorption.^13^ Lack of SOST, which leads to sclerosteosis, is characterized by high bone mass.^16^ While the overexpression of sclerostin decreased bone strength.^17^

A discovery that osteocytes were important for the regulation of osteoblasts and osteoclasts provided a new way to study the mechanism of periprosthetic osteolysis. Lohmann et al.^18^ demonstrated that the addition of UHMWPE to MLO‐Y4 osteocytes in vitro significantly raised levels of prostaglandin E2 and nitric oxide. Kanaji et al.^19^ showed that metal particles also significantly caused apoptosis of osteocytes, and was associated with secretion of TNF‐α. Zhang et al.^20^ found that wear particles of tricalcium phosphate could cause dysfunction in MLO‐Y4 osteocytes, mediated by apoptosis and Akt signal inactivation. Wang et al.^21^ demonstrated that conditioned medium from osteocytes challenged with Ti‐alloy particle promoted osteoclast formation. We found that osteolysis on the skull surface of mice increased with a high level of sclerostin after treated with Ti particles. After reduction of SOST, number of lytic pores on the skull surface decreased.^22^ These studies suggested that osteocytes play an important role in osteolysis induced by Ti particles. However, the regulation of osteocyte injured by wear debris on osteoblast or osteoclast remains largely unknown.

In this study, we challenged osteocytic cell line MLO‐Y4 with commercial titanium (Ti) particles, and investigated their direct biological effects on MLO‐Y4 cells. Furthermore, we studied the effects of Ti‐induced osteocytic alterations on MC3T3‐E1 osteoblastic cell using two co‐culture systems in vitro. We found MLO‐Y4 cells treated with Ti particles inhibited osteoblastic differentiation mostly via direct cell‐to‐cell contact. During this process, sclerostin induced by Ti particles plays an important role on the regulation of osteocyte over osteoblast.

## 2 MATERIALS AND METHODS

### 2.1 Detection and preparation of Ti particle

Pure Ti particles were purchased from Johnson Matthey (catalogue #00681; Ward Hill). Ninety percent of Ti particles were <10.0 μm in size and the average diameter was 5.34 μm, according to the manufacturer. Such commercially available particles have been proved the effectiveness of mimic wear debris obtained from joint aseptic loosening in our previous study.^10^

The particles were prepared as previously described.^10^ The endotoxin‐free detection of Ti particles was performed and confirmed using a QCL‐1000 kit (Biowhittaker). Ti particles were mixed with PBS at concentration of 10 mg/ml, then the stock solution was sonicated and diluted with medium to 0.1 or 1 mg/ml for experiments.

### 2.2 Cell culture and treatments

Osteocytic cell line MLO‐Y4 and osteoblastic cell line MC3T3‐E1 were obtained from the Chinese Academy of Sciences Cell Bank. MLO‐Y4 cells were cultured in modified essential medium (α‐MEM) containing 10% foetal bovine serum (Gibco), 1% penicillin and streptomycin. The MLO‐Y4 cells were separately incubated with Ti particles at concentrations of 0 (control), 0.1 and 1.0 mg/ml after 24 h of seeding; fresh medium was supplied every 3 days. The time of addition of Ti particles to cells was designated as day 0.

The model for co‐culture of MLO‐Y4 and MC3T3‐E1 in vitro was constructed using the Millicell Culture Insert Plate (Millipore), which is comprised of the membrane perforated with 1‐μm pores, as previously described.^23^ Basal medium for co‐culture experiments was comprised of α‐MEM supplemented with 10% FBS, 1% penicillin and 1% streptomycin. The osteogenic differentiation medium consisted of 10% FBS, 10 mM of β‐glycerophosphate and 50 μg/ml of ascorbic acid (Sigma‐Aldrich). Using co‐cultures, we detected the osteoblastic markers of MC3T3 cells and evaluated osteoblastic differentiation. Three co‐culture models were as follows.

**FIGURE 1:**
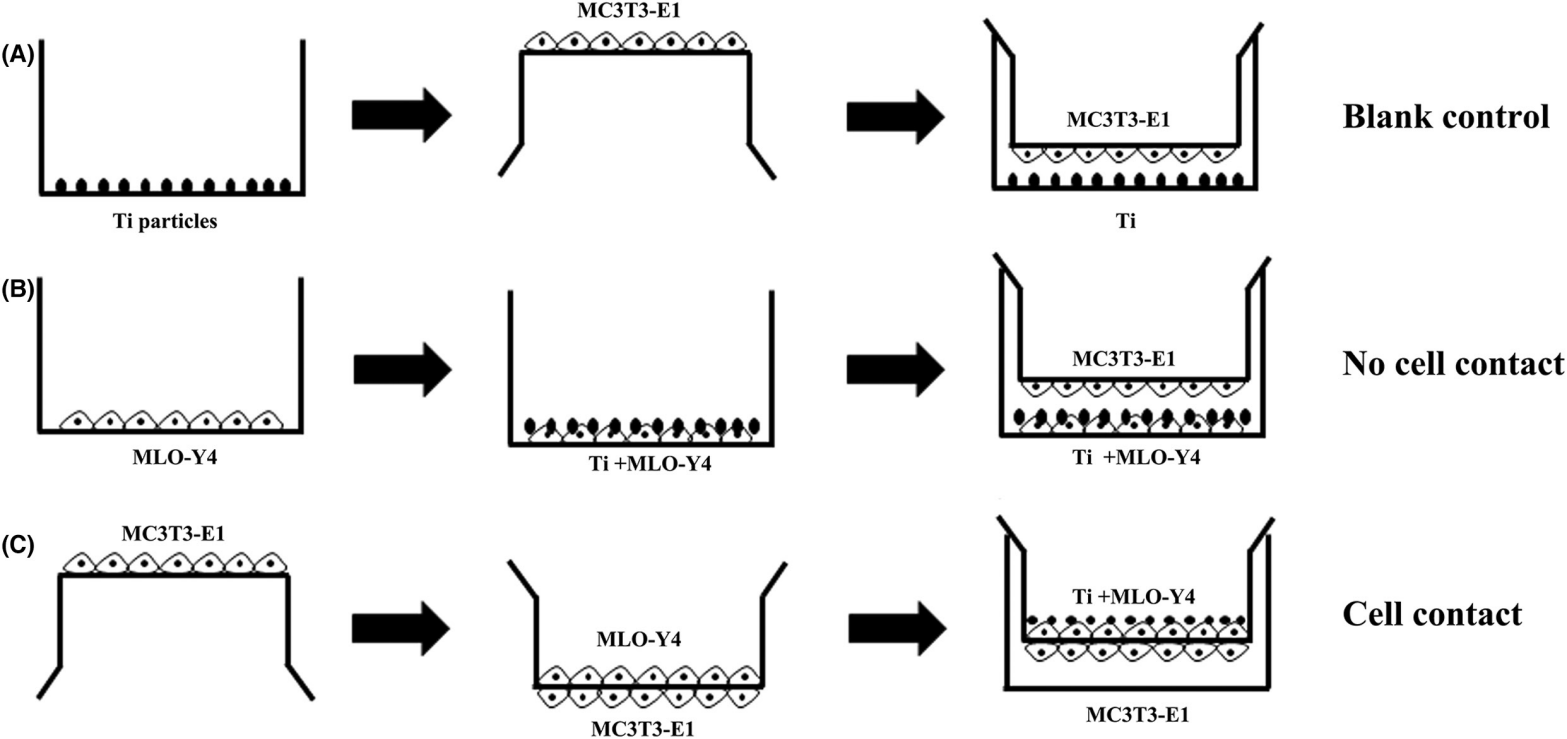
Schematic diagram showing seeding of the two cell types in the different co‐culture systems. (A) ‘Blank control’ model: MC3T3‐E1 osteoblastic cells were seeded on the basal surface of the membrane (bottom side of insert), and Titanium (Ti) particles were then seeded on the bottom of the wells. (B) ‘No Cell Contact’ model: MC3T3‐E1 cells were seeded on the basal surface of the membrane, and MLO‐Y4 osteocytic cells were seeded on the bottom of the wells. The two cell types could not come into direct contact. MLO‐Y4 cells were treated with Ti particles. (C) ‘Cell Contact’ model: MC3T3‐E1 cells were seeded on the basal surface of the membrane and MLO‐Y4 cells were then seeded on the apical surface of the membrane. The two cell types were permitted to come into direct contact. MLO‐Y4 cells were treated with Ti particles

For the blank control group, there were no MLO‐Y4 cells seeded. Ti particles at concentrations of 0 or 1.0 mg/ml were diluted with 2 ml of basal medium and added to the bottom of the six‐well plate. Then, insert plates was inverted, and the basal surface of the membrane (bottom side of insert) was seeded with MC3T3‐E1 cells at the density of 1 × 10^4^ cells/cm^2^ in 500 μl of basal medium and incubated for 6 h at 37°C to permit cellular adhesion. Ti particles were seeded into six‐well plates. After Ti particles deposited to the bottom of the plate about 6 h later. Millcell culture inserts, seeded with MC3T3‐E1 cells as mentioned, were inverted and inserted into six‐well plates (Figure 1A). The time of addition of the Ti particles was designated as day 0. The medium was replaced with osteogenic medium after 3 days and was subsequently changed every 3 days.

For the ‘no cell contact’ model, MLO‐Y4 cells were firstly seeded directly at the density of 2 × 10^3^ cells/cm^2^ on the bottom of the six‐well plate with 2 ml of basal medium and incubated overnight. After the medium removed, Ti particles at concentrations of 0 or 1.0 mg/ml, were diluted with basal medium and added to the bottom of the six‐well plate. Then, insert plates was inverted, and the basal surface of the membrane was seeded with MC3T3‐E1 cells at the density of 1 × 10^4^ cells/cm^2^ in 500 μl of basal medium and incubated for 6 h at 37°C to permit cellular adhesion. After 6 h, insert was inserted into Millicell six‐well tissue culture plate (Figure 1B). All other conditions were same as described for the blank control.

In the ‘cell contact’ co‐culture model, insert plate was inverted, and the basal surface of the membrane was seeded with MC3T3‐E1cells at the density of 1 × 10^4^ cells/cm^2^ in 500 μl of basal medium and incubated for 6 h. Then insert was inserted into Millicell six‐well tissue culture plate containing 1 ml of basal medium. MLO‐Y4 cells were seeded at the density of 2 × 10^3^ cells/cm^2^ on the apical side of the membrane (top side of insert) with 1 ml of basal medium and incubated overnight. The MLO‐Y4 cells on the upper side were treated with Ti particles at concentrations of 0 or 1.0 mg/ml, diluted with basal medium (Figure 1C). All other conditions were same as described for the blank control.

### 2.3 Real‐time PCR quantification of mRNA expression of SOST

Similarly, MLO‐Y4 cells were seeded into six‐well plates at a density of 1 × 10^4^ cells/cm^2^ and incubated with different concentrations of Ti particles for 24 and 48 h. Trizol (Invitrogen) was used to extract total RNA from MLO‐Y4 cells. One microgram of total RNA was reverse‐transcribed to generate first‐strand DNA (Thermo) and used as a template for PCR. Quantitative real‐time PCR was performed with SYBR Green I (Roche) Kit, according to the manufacturer's instructions. Glyceraldehyde 3‐phosphate dehydrogenase (GAPDH) was used as an internal control. The primers sequences were as follows: SOST (Forward, 5′–ATCCCAGGGCTTGGAGAGTA–3′; Reverse, 5′–ACATCTTTGGCGTCATAGGG–3′), and GAPDH (Forward 5′–GTGTGGTCACTATTTGCCTGTC–3′; Reverse 5′–AAGCAGTTGGTGGTGCAGGA–3′). The PCR profile began with a temperature of 95°C for 5 min to activate the DNA polymerase, followed by 35 cycles of denaturation (95°C for 30 s) and annealing (57°C for 30 s) at, extension (72°C for 1 min). The relative amount of SOST mRNA expression compared with GAPDH was expressed as a fold change, which was calculated relative to the control group using the comparative *C*_t_ ($2^{-\Delta \Delta C_{t}}$).

### 2.4 Western‐blot analysis for protein expression

#### 2.4.1 The protein expression of sclerostin, β‐catenin, connexin 43 (Cx43), cleaved caspase‐3, Bax, Bcl‐2, IL‐6 and TNF‐α in MLO‐Y4 osteocytes

MLO‐Y4 cells were seeded into six‐well plates for the different experimental intervention. Cells were washed twice with PBS, treated with lysis buffer and put on ice for 20 min, then centrifuged at 21,130 *g* for 30 min. The supernatant was collected and the protein concentration was measured using a BCA protein assay kit (Beyotime, P0010). About 30 μg of protein samples were separated by 10% SDS‐PAGE and electro‐blotted onto nitrocellulose membranes. After blocking with 5% bovine serum albumin (Sangon Biotech, 4240GR100) for 3 h at room temperature, membranes were incubated with a 1:1000 dilution of primary monoclonal antibodies against sclerostin (Abcam, 86465), β‐catenin (dephosphorylated form, CST, 8480T) and Cx43 (Abcam, ab11370) overnight at 4°C. After washing four times with TBST (Tris‐buffered saline with Tween), the membranes were incubated with horseradish peroxide‐conjugated goat anti‐Rat IgG (Multisciences, GRT007), goat anti‐rabbit IgG (Multisciences, GAR007) for 60 min at 25°C, finally wash the membranes three times for 10 min each time with TBST. Protein signals were illuminated with electrochemiluminescence, and analysed with GIS image analysis system.

In order to detect the proteins related to cell apoptosis, about 50 μg of protein samples were separated by 10% SDS‐PAGE and membranes were incubated with a 1:1000 dilution of primary antibodies against Cleaved caspase‐3 (CST, 9664), Bax (Proteintech, 60267) and Bcl‐2 (Proteintech, 60178) overnight at 4°C. For the detection of IL‐6 and TNF‐α, about 50 μg of protein samples were separated by 12.5% SDS‐PAGE and electro‐blotted onto nitrocellulose membranes. Membranes were incubated with a 1:1000 dilution of primary monoclonal antibodies against IL‐6 (Abcam, 9324) and TNF‐α (ProteinTech, 17590‐1‐AP) overnight. Protein analysis procedures were performed as described above.

#### 2.4.2 The proteins expression of ALP, RUX2 and OCN in MC3T3‐E1 cells

In the co‐culture models, MC3T3‐E1 cells were collected for the detection of alkaline phosphatase (ALP) and Runx2 on 7 days and for the detection of osteocalcin (OCN) on 21 days separately. MC3T3‐E1 cells were collected using cell scrapers not trypsin. Membranes were incubated with a 1:1000 dilution of primary antibodies against ALP (Abcam, 95462), Runx‐2 (Abcam, 76956) and OCN (Santa Cruz, 365797) overnight at 4°C. Protein analysis procedures were performed as described above.

### 2.5 Cellular apoptosis detected by flow cytometry

MLO‐Y4 cells were seeded into six‐well plates at a density of 1 × 10^4^ cells/cm^2^ overnight and treated separately with Ti particles (0, 0.1 and 1.0 mg/ml) for 24 and 48 h. Apoptosis of MLO‐Y4 cells was evaluated by flow cytometry. The cells and the culture media were collected separately. MLO‐Y4 cells were digested by 0.25% trypsin without EDTA. MLO‐Y4 cells and the media were centrifuged separately at 100 *g* for 5 min and the supernatant was discarded. Two parts of sediment were resuspended and mixed up with 400 μl of binding buffer and 5 μl of Annexin V‐FITC (BD Biosciences) were added; cells were incubated in the dark at room temperature for 15 min, and 5 μl of propidium iodide (PI) were added. The cells were analysed using a FACS Calibur flow cytometer (BD Biosciences). In each case, 10,000 cells were subjected to flow cytometric analysis. After SOST silence and overexpression, apoptosis of MLO‐Y4 cells was detected again by flow cytometry and the procedures were performed as described above.

### 2.6 Immunofluorescence staining for sclerostin, β‐catenin, Cx43 and cleaved‐caspase‐3

MLO‐Y4 cells were washed with PBS, fixed with 4% paraformaldehyde for 10 min and permeabilized with 0.1% Triton X‐100 for 5 min. Then, the cells were incubated with a rat anti‐mouse primary antibody against sclerostin (Abcam, 86465) mouse anti‐mouse primary antibody against β‐catenin (Proteintech, 66379) and rabbit anti‐mouse primary antibody against Cx43 (Abcam, ab11370) for 12 h at 4°C. Following three washes in PBST, the cells were incubated with goat anti‐rat Alexa Fluor®‐594 (Abcam, ab150160) secondary antibody, goat anti‐mouse Alexa Fluor®647 (Abcam, ab150115) secondary antibody and goat anti‐rabbit Alexa Fluor®488 (Abcam, ab150077) secondary antibody for 1 h in a dark room. Then cells were washed in PBST and nuclei were stained with DAPI for 5 min. These stained cells were observed under a laser‐scanning confocal microscope LSM880 (Zeiss) with an excitation wavelength of 594, 647 and 488 nm.

To detect cleaved‐caspase‐3, MLO‐Y4 cells were incubated with rabbit anti‐mouse primary antibody against cleaved‐caspase‐3 (CST, 9664T) and then incubated with goat anti‐rabbit Alexa Fluor®488 (Abcam, ab150077) secondary antibody. MLO‐Y4 cells were observed with an excitation wavelength of 488 nm.

### 2.7 Sclerostin, IL‐6 and TNF‐α in the co‐culture media detected with ELISA

Media from the co‐culture models of ‘no cell contact’ and ‘cell contact’ were collected on day 3 and stored at −80°C. The level of sclerostin, IL‐6 and TNF‐α was quantified using enzyme‐linked immunosorbent assay (ELISA) kits (R&D Systems, MSST00; Elabscience, E‐EL‐M0044c; Elabscience, E‐EL‐M3063). All procedures were performed according to the manufacturer's instructions.

### 2.8 Transfection for SOST silencing or overexpression

SOST of MLO‐Y4 osteocytic cells was silenced using short hairpin shRNA lentiviral particles (Sigma‐Aldrich Chemical Co), following the manufacturer's instructions. Briefly, after searching the sequence of the SOST gene in GenBank, the interference sequence SOST‐shRNA was designed using RNA interference from Invitrogen. The shRNA sequences were artificially synthesized by Sangon Biotech as follows: 5′–GACAGCATATCTTACATTAAA−3′. Cells were infected with lentiviral particles carrying either scrambled or SOST‐ specific shRNA. The efficiency of deletion was determined by measuring SOST mRNA expression and protein by real‐time PCR and by Western blotting, separately. SOST overexpression of MLO‐Y4 cells were manipulated with the same as SOST silencing.

### 2.9 Alkaline phosphatase activity and staining

Alkaline phosphatase activity in the supernatant from three different co‐culture systems was measured on day 7 after challenged with/without Ti particles. In preparation for this assay, medium was collected and centrifuged to remove cell debris and Ti particles. ALP activity was assayed using an Alkaline Phosphatase Assay Kit (Sigma‐Aldrich). In brief, MC3T3‐E1 cells were collected using cell scrapers not trypsin. Treated with lysis buffer (without PMSF), and put on ice for 20 min, then centrifuged at 21,130 *g* for 15 min, collected the supernatant and mixed the centrifuged medium. The substrate solution pNPP prepared according to the instructions was added and incubated the 96‐well plates in the dark for 30 min at room temperature. After NaOH terminated the reaction, the absorbance was read at 405 nm.

Three different co‐culture models were maintained as described above. ALP staining was performed on day 7 after challenge with Ti particles. MC3T3‐E1 cells were stained using an Alkaline Phosphatase Stain Kit (Jiancheng). In brief, cells were fixed in methanol and added with 5‐bromo‐4‐chloro‐3‐indolyl phosphate plus nitroblue tetrazolium chloride in Tris–HCl, NaOH and MgCl_2_ successively, then incubated at room temperature for 2 h in the dark.

### 2.10 Mineralized nodule staining and detection of Ca^2+^ levels

Three different co‐cultures were maintained as described above. Mineralization of MC3T3‐E1 cells was monitored on day 21 by visualization with alizarin red S (Sigma‐Aldrich) staining. Briefly, after 3 weeks, MC3T3‐E1 osteoblastic cells were washed with PBS prior to fixation with 70% ethanol, and stained with 1% (w/v) alizarin red solution (pH 4.3) at room temperature. To quantify the amount of alizarin red, the deposition was dissolved in 10% (w/v) cetylpyridinium chloride prepared in double‐distilled H_2_O (ddH_2_O) and quantified by measuring the OD value at 562 nm.

### 2.11 Statistical analysis

Data were analysed with spss version 17.0. All data are expressed as the mean ± standard deviation, and each assay was repeated independently three times. The difference of statistical analysis was evaluated by One‐way analysis of variance (anova) and post‐hoc multiple comparisons. A difference was considered significant if *p* < 0.05.

## 3 RESULTS

### 3.1 Ti particles increased SOST/sclerostin expression and decreased β‐catenin expression of MLO‐Y4 cells, and reduction of SOST improved β‐catenin expression

**FIGURE 2:**
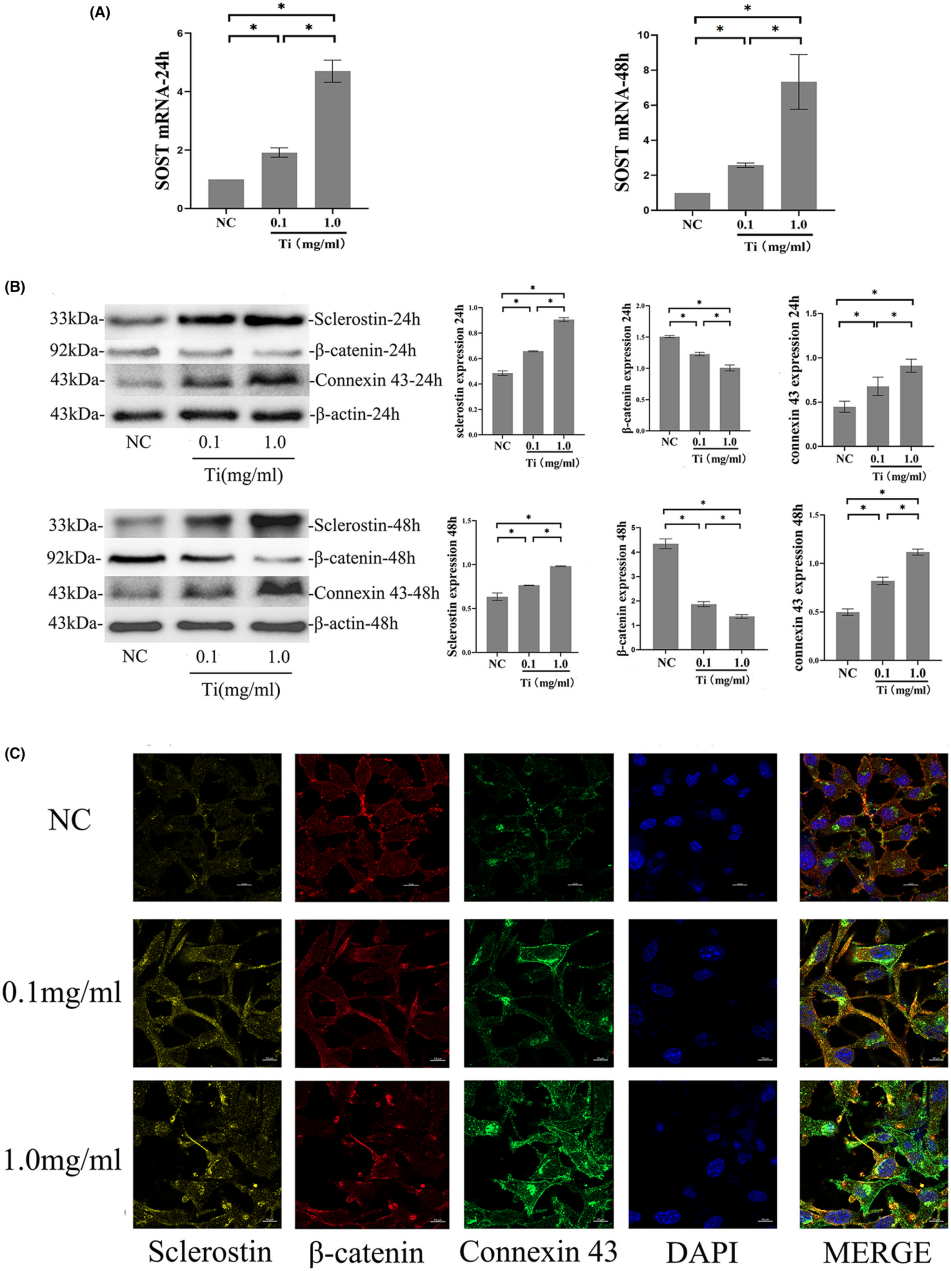
The effects of Titanium (Ti) particles on MLO‐Y4 cells. (A) mRNA expression of SOST in MLO‐Y4 cells treated with Ti particles for 24 and 48 h. At 24 and 48 h, treatment with 0.1 and 1.0 mg/ml of Ti particles significantly increased SOST mRNA expression compared with the control group (*p < 0.05). The group treated with 1.0 mg/ml of Ti particles also differed significantly from the group treated with 0.1 mg/ml (*p < 0.05). Ti particles increased SOST expression of MLO‐Y4 cells in a dose‐dependent manner. The relative amount of SOST mRNA expression compared with GAPDH was expressed as a fold change, which was calculated relative to the control group using the comparative *C*_t_ ($2^{-\Delta \Delta C_{t}}$). (B) Sclerostin, β‐catenin and connexin 43 protein expression of MLO‐Y4 cells treated with the different concentration of Ti particles for 24 and 48 h. Protein levels of sclerostin, β‐catenin and connexin 43 were detected by western blot and normalized to β‐actin. Treatment with 0.1 or 1.0 mg/ml of Ti particles significantly increased sclerostin and connexin 43 expression compared with the control group at 24 and 48 h (*p < 0.05). β‐catenin expression was significantly lower in the 0.1 mg/ml group and the 1.0 mg/ml group than the control group at 24 and 48 h (*p < 0.05). Ti particles increased the expression of sclerostin and connexin 43 but decreased the expression of β‐catenin in a dose‐dependent manner. Error bar indicates the standard deviation of 3 repeats. (C) Sclerostin, β‐catenin and connexin 43 expression in MLO‐Y4 cells by immunofluorescence analysis at 48 h. Blue, DAPI nuclear staining; Yellow, sclerostin; Red, β‐catenin; Green, Connexin 43; The expression of sclerostin and connexin 43 in MLO‐Y4 cells treated by Ti particles increased compared with that in MLO‐Y4 cells without Ti particles. Sclerostin not only accumulated in the cytoplasm but also in long dendritic processes of MLO‐Y4 cells. β‐catenin in MLO‐Y4 cells treated by Ti particles decreased compared with that in MLO‐Y4 cells without Ti particles. NC, the negative control group (without Ti particles treatment); mRNA, messenger RNA; DPAI, 4, 6‐diamino‐2‐phenyl indole

We examined expression of the osteocytic marker, SOST/sclerostin. The data showed that Ti particles of 0.1 mg/ml group and 1.0 mg/ml group at 24 and 48 h significantly increased SOST mRNA expression compared with the control (*p* < 0.05) and SOST expression increased with the increasing of Ti particles concentration (Figure 2A). To verify SOST changes induced by Ti particles, we further analysed the protein expression of sclerostin. Protein expression was generally consistent with mRNA expression, and Ti particles with the concentration of 0.1 and 1.0 mg/ml resulted in a clear increase in sclerostin protein levels at 24 and 48 h (*p* < 0.05). While protein expression of β‐catenin in the group treated with Ti particles with the concentration of 0.1 and 1.0 mg/ml decreased significantly at 24 and 48 h compared with the group without Ti particles (*p* < 0.05) (Figure 2B). Through immunofluorescent staining, we verified again that sclerostin accumulated in the cytoplasm and long dendritic processes of osteocytic cells increased significantly in the Ti‐treated group compared with the control. Instead, β‐catenin expression decreased significantly in the Ti‐treated group compared with the control (Figure 2C).

**FIGURE 3:**
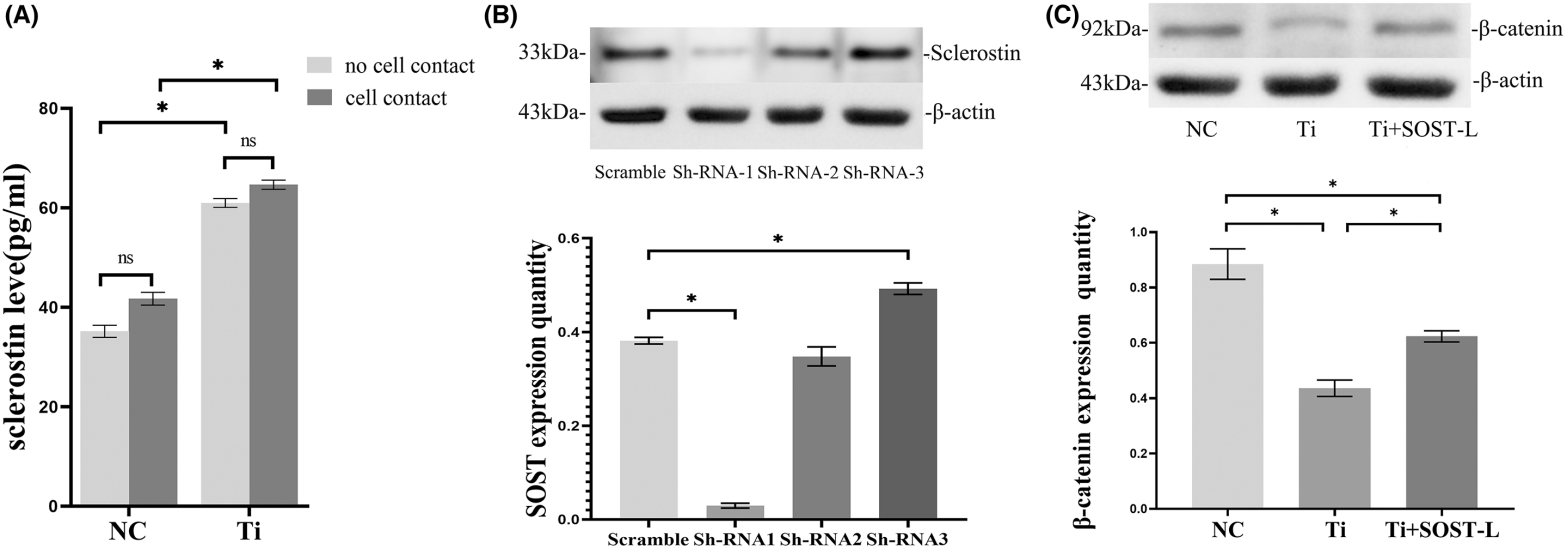
(A) Sclerostin level in media of the different co‐culture models on day 3. Sclerostin level in media both went up after treated with 1.0 mg/ml Ti particle in the co‐cultures of ‘no cell contact’ and ‘cell contact’. There was significant difference between the group with Ti group and the group without Ti particles (*p < 0.05). But there were no significant difference in sclerostin level at the same concentration of Ti particles between ‘no cell contact’ model and ‘cell contact’ model. (B) Validation and screen of SOST knockdown. After lentiviral transfection, the efficiency of SOST‐shRNA was detected by measuring sclerostin protein expression using western blotting. Sclerostin in the shRNA‐1 group has a significant decrease compared with that in the scramble group (*p < 0.05). The first sequence of shRNA (shRNA‐1) was chosen for experiments of SOST silence. (C) Effects of SOST silence on the expression of β‐catenin. Protein levels of β‐catenin were detected by western blot. After exposed to 1.0 mg/ml Ti particles for 48 h, β‐catenin level was lower than the group without Ti particles (*p < 0.05). After SOST silence, the expression of β‐catenin has a markable increasing, even if MLO‐Y4 cells were treated with Ti particles (*p < 0.05). Error bar indicates the standard deviation of 3 repeats. shRNA: short hairpin RNA; NC, the negative control group (without Ti particles); Ti, the group treated with 1.0 mg/ml Ti particles; Ti + SOST‐L, the group treated with Ti particles after SOST silence, in which SOST was low‐expression

To explore the effects of osteocyte on osteoblast, we co‐cultured MLO‐Y4 cells and MC3T3‐E1 cells. Sclerostin in media was detected using ELISA kits in two co‐culture model of ‘no cell contact’ and ‘cell contact’ on day 3. Sclerostin level went up in the Ti‐treated group (1.0 mg/ml) compared with the group free from Ti particles (*p* < 0.05). There was no significant difference on sclerostin level in the groups with Ti particles between ‘no cell contact’ model and ‘cell contact’ model (Figure 3A).

To verify further the effects of SOST/sclerostin, we silenced SOST. For SOST silence, three interference sequences of SOST‐shRNA were designed and identified. The first shRNA sequences significantly reduced the expression of sclerostin (Figure 3B), and we chose the first shRNA for next experiments. After SOST silencing, β‐catenin protein levels increased significantly (*p* < 0.05), even though MLO‐Y4 had been treated with Ti particles (Figure 3C).

### 3.2 Ti particles induced apoptosis of MLO‐Y4 cells, SOST silence decreased apoptosis and alleviated the increase of apoptosis caused by Ti particles

To assess MLO‐Y4 cells changes induced by Ti particles, we measured apoptosis of MLO‐Y4 cells by flow cytometry. Incubated with Ti particles for 24 or 48 h, the apoptosis of MLO‐Y4 cells increased rapidly, and was dependent on the Ti particle concentration (Figure S1). After 24 h, the percentage of apoptotic cells increased in both two groups treated with Ti particles, apoptotic cells in the 0.1 mg/ml group and the 1.0 mg/ml group differed significantly from the control (*p* < 0.05). The percentages of apoptosis remained high in the 0.1 and 1.0 mg/ml groups and differed significantly from the control at 48 h (*p* < 0.05). Ti particles induced apoptosis of MLO‐Y4 cells in a dose‐dependent manner. To explore the relationship between SOST and apoptosis, the apoptosis of MLO‐Y4 cells was evaluated again after SOST silenced or overexpressed. The protein expression of Bax and Bcl‐2, which were the markers of apoptosis, was detected. The result showed that compared with the control group, the ratio of Bax/Bcl‐2 decreased when SOST was low‐expression and the ratio of Bax/BCL‐2 increased after sclerostin overexpressed (*p* < 0.05) (Figure S2A). Flow cytometry results also showed that compared with the control group, the apoptosis of MLO‐Y4 cells decreased significantly after SOST silence (*p* < 0.05). Inversely, the apoptosis increased significantly after SOST overexpression (*p* < 0.05) (Figure S2B).

**FIGURE 4:**
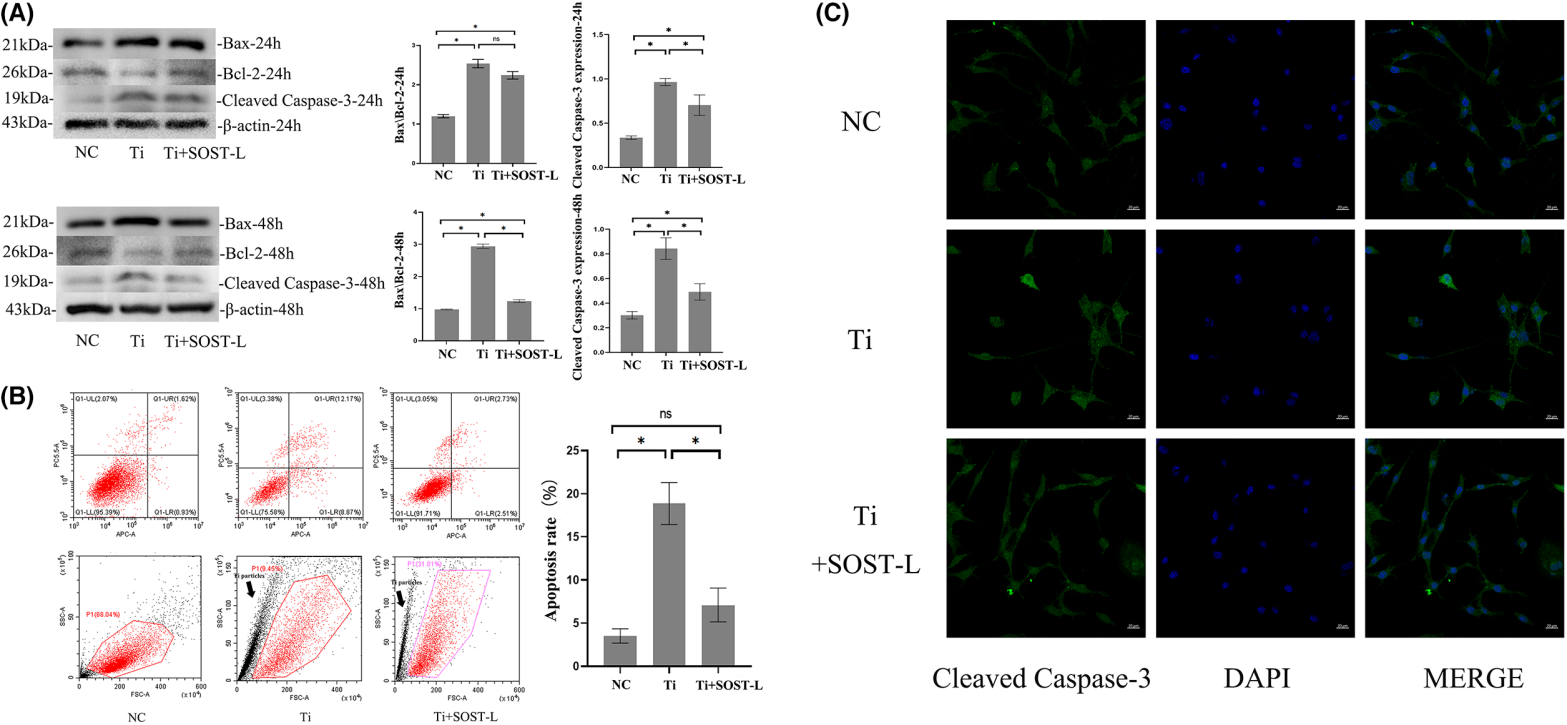
Ti particles induced apoptosis of MLO‐Y4 cells, SOST silence decreased apoptosis and alleviated the increase of apoptosis caused by Ti particles. (A) The detection for proteins related to apoptosis by western‐blot. After treatment with 1.0 mg/ml Ti particles for 24 and 48 h, protein expressions of Bax, Bcl‐2 and cleaved‐caspase‐3, were detected. After Ti particle intervention, the ratio of Bax/ Bcl‐2 and cleaved caspase‐3 expression were significantly increased compared with the group free from Ti particles (*p* < 0.05) (Figure 4A). While the ratio of Bax/Bcl‐2 and cleaved caspase‐3 expression decreased significantly even treated with Ti particles when SOST was silenced (*p* < 0.05). (B) Flow cytometric analysis of Ti particle‐induced apoptosis in MLO‐Y4 cells by Annexin V/PI staining. After exposed to 1.0 mg/ml Ti particles for 48 h, cells were stained with FITC‐conjugated Annexin V. Apoptosis of cells was quantified by flow cytometry. Compared with the control group, Ti particles increased the percentage of apoptotic cells (*p < 0.05). Scattered points near the *Y*‐axis represented Ti particles, marked with black arrow. Compared with Ti‐treated group, the apoptosis of MLO‐Y4 cells treated with Ti particle decreased significantly after SOST silence (*p* < 0.05). (C). Immunofluorescence stain showed the expression of cleaved‐caspase‐3 in MLO‐Y4 cells. After exposed to 1.0 mg/ml Ti particles for 48 h, cells were stained with immunofluorescence. Green, cleaved‐caspase‐3; Blue, DAPI nuclear staining. Compared with the control group, Ti particles increased the expression of cleaved‐caspase‐3 in MLO‐Y4 cells. While the expression of cleaved caspase‐3 in MLO‐Y4 cells decreased after SOST silence compared with Ti‐ treated group. NC, the negative control group (without Ti particles); Ti, the group treated with 1.0 mg/ml Ti particles; Ti + SOST‐L, the group treated with Ti particles after SOST silence, in which SOST was low‐expression. FITC, fluorescein isothiocyanate; PI, Propidium iodide. Error bar indicates the standard deviation of 3 repeats

We detected further the effect of SOST silence on apoptosis of MLO‐Y4 cells with the treatment of Ti particle. The protein expression of BAX, Bcl‐2 and cleaved caspase‐3 was detected. After Ti particle intervention, the ratio of Bax/Bcl‐2 and cleaved caspase‐3 expression were significantly increased compared with the group free from Ti particles (*p* < 0.05) (Figure 4A). The ratio of Bax/Bcl‐2 and cleaved caspase‐3 expression decreased significantly in Ti+SOST‐L group compared with Ti‐treated group (*p* < 0.05) (Figure 4A). Flow cytometry results also showed that compared with Ti‐treated group, the apoptosis of MLO‐Y4 cells decreased significantly with the existence of Ti particle after SOST silence (*p* < 0.05) (Figure 4B). By immunofluorescence staining, we found the results similar to the flow cytometry results that the expression of cleaved caspase‐3 in MLO‐Y4 cells increased significantly with the treatment of Ti particles, while the expression of cleaved caspase‐3 in MLO‐Y4 cells decreased significantly after SOST silence compared with Ti‐treated group (Figure 4C).

### 3.3 Ti particles increased the expression of IL‐6 and TNF‐α, and SOST silence decreased the expression of IL‐6 and TNF‐α

**FIGURE 5:**
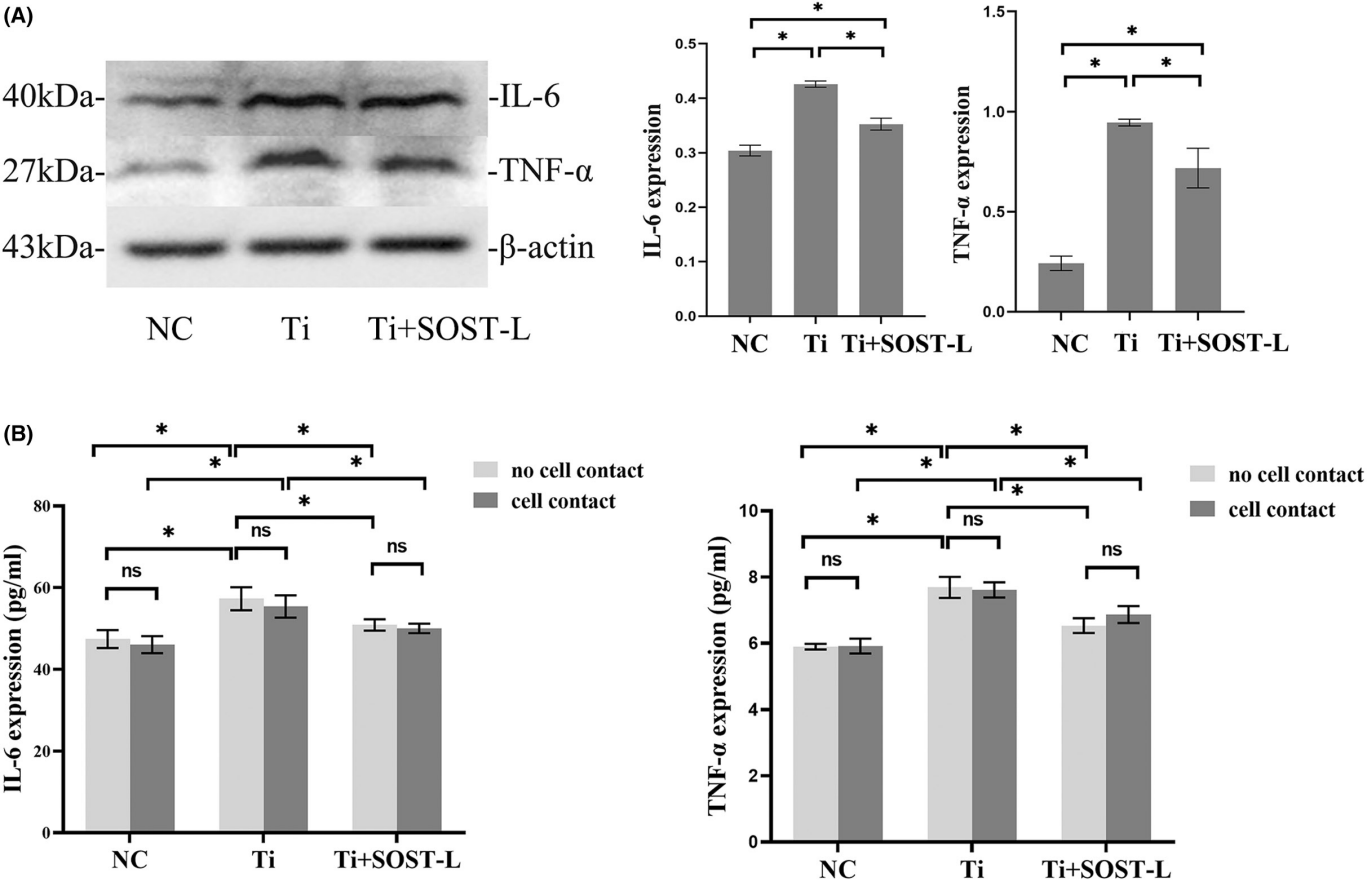
Ti particles increased the expression of IL‐6 and TNF‐α, and SOST silence decreased the expression of IL‐6 and TNF‐α. (A) Protein expression of IL‐6 and TNF‐α in MLO‐Y4 cells was detected by western blot. After exposed to 1.0 mg/ml Ti particles for 48 h, the expression of IL‐6 and TNF‐α was higher than that of control group (*p < 0.05). The expression of IL‐6 and TNF‐α in MLO‐Y4 cells decreased significantly after SOST silence, even if MLO‐Y4 cells were treated with Ti particles (*p < 0.05). (B) The levels of TNF‐α and IL‐6 in the medium from two co‐culture models were assayed by ELISA on day 3. TNF‐α and IL‐6 level in media both went up after treated with 1.0 mg/ml Ti particle in the co‐cultures of ‘no cell contact’ and ‘cell contact’. There was no significant difference in the level of IL‐6 and TNF‐α between ‘cell contact’ model and ‘no cell contact’ model at the same concentration of titanium particles. The level of IL‐6 and TNF‐α in the Ti‐treated group was significantly highter than NC group in both ‘cell contact’ and ‘no cell contact’ model (*p < 0.05), and the level of IL‐6 and TNF‐α were decreased after SOST silence, even if MLO‐Y4 cells were treated with Ti particle (*p < 0.05). NC, the negative control group (without Ti particles); Ti, the group treated with 1.0 mg/ml Ti particles; Ti + SOST‐L, the group treated with Ti particles after SOST silence, in which SOST was low‐expression. ns, no significance

To clarify the effects of Ti particles on the expression of inflammatory factors in MLO‐Y4 cells, we examined the protein expression of IL‐6 and TNF‐α. The results showed that, the expression of IL‐6 and TNF‐α increased significantly in the treatment with Ti particles compared with the control group (*p* < 0.05). While the expression of the IL‐6 and TNF‐α decreased significantly after SOST silence, even if MLO‐Y4 cells were treated with Ti particles (*p* < 0.05) (Figure 5A). Meanwhile, we detected the level of IL‐6 and TNF‐α in the medium by ELISA in the two different co‐culture model, ‘no cell contact’ and ‘cell contact’. The results showed that at the same concentration of Ti particles, there was no significant difference in the level of IL‐6 and TNF‐α between ‘cell contact’ model and ‘no cell contact’ model. However, the expression of IL‐6 and TNF‐α in the group treated with Ti particles was significantly higher than that without Ti particles in the same co‐culture model (*p* < 0.05), and IL‐6 and TNF‐α were decreased after SOST silence, even if MLO‐Y4 cells were treated with Ti particle (*p* < 0.05) (Figure 5B).

### 3.4 Ti‐treated MLO‐Y4 cells inhibited the osteoblastic differentiation of MC3T3‐E1 cells mostly through direct cell–cell contact

To ascertain the effects of Ti‐treated MLO‐Y4 cells on MC3T3‐E1 cells, ALP activity and ALP staining were performed on day 7, mineralized nodule staining and colorimetrical quantitative analysis of alizarin red, which detects Ca^2+^ levels, were performed on day 21. The MLO‐Y4 cells in the ‘no cell contact’ and ‘cell contact’ co‐culture models were simultaneously challenged with Ti particles. As the blank control, MC3T3‐E1 cells were cultured without MLO‐Y4 and Ti particles were added to the bottom of the six‐well plate. In the blank control, ALP activity, ALP staining, Ca^2+^ levels and the number of mineralized nodules did not differ significantly in the groups with/without Ti Particles.

**FIGURE 6:**
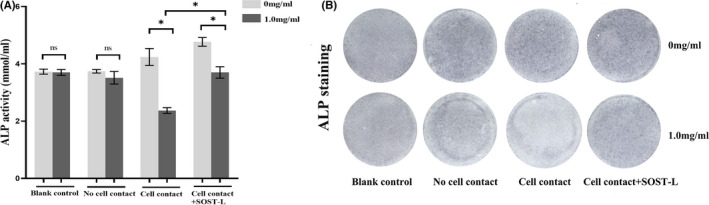
Effects of MLO‐Y4 changes induced by Ti particle on ALP activity and ALP staining of MC3T3‐E1 cells in the different co‐culture models on day 7. (A) Data showing ALP activity are expressed as mean ± standard deviation. In the ‘blank contact’ model and the ‘no cell contact’ model, the ALP activity of the group treated with 1.0 mg/ml Ti particles did not differ significantly from that of the group without Ti particles. In the ‘cell contact’ model, ALP activity of the group with Ti particles significantly decreased compared with the group without Ti particles (*p < 0.05). After SOST silencing, ALP activity increased, even if MLO‐Y4 cells were treated with Ti particles. The level of ALP had significant difference after and before SOST silence (*p < 0.05). (B) In the ‘blank control’ and ‘no cell contact’ models, no significant differences in ALP staining were observed in the group with/without Ti particles. ALP staining in the ‘cell contact’ model showed fewer positive cells in the 1.0 mg/ml groups compared with the group free from Ti particles. After SOST silencing, ALP positive cells increased and the difference of ALP positive cells was significant after and before SOST silence. ALP, Alkaline phosphatase

**FIGURE 7:**
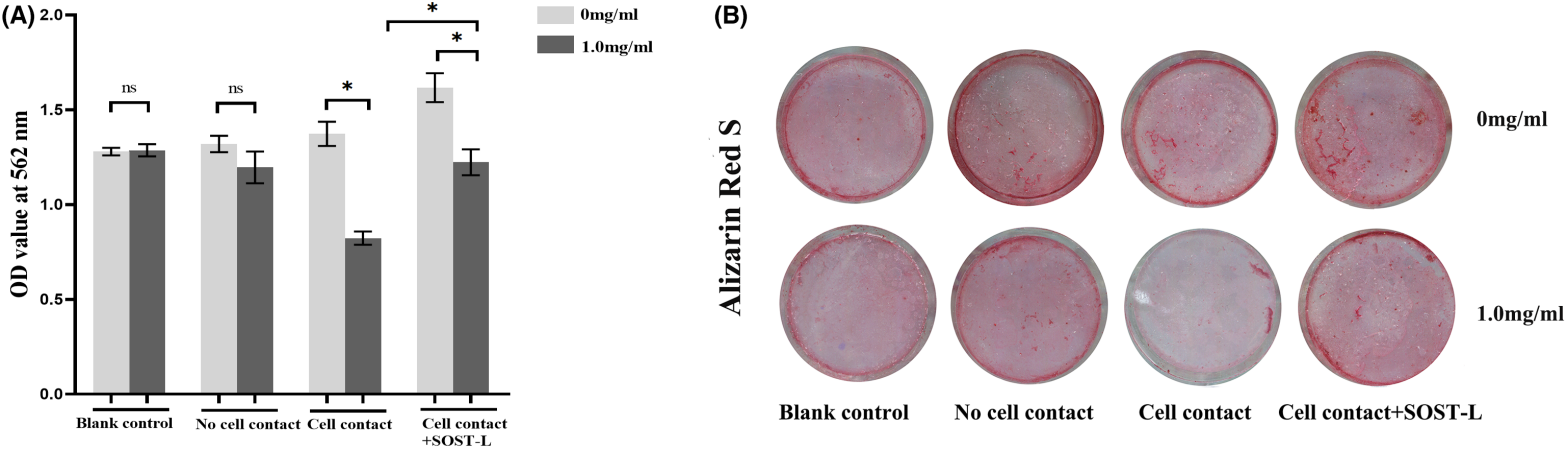
Effects of Ti particle‐induced changes in MLO‐Y4 cells on mineralization of MC3T3‐E1 cells in two co‐culture models on day 21. (A) Data showing in the ‘Blank Contact’ model and the ‘no cell contact’ model, no significant differences in Ca^2+^ levels were observed in the group with/without group. In the ‘cell contact’ model, quantitative results of Ca^2+^ levels differed significantly between the group with Ti particles and the group without Ti particles (*p < 0.05). After SOST inhibition, Ca^2+^ levels increased. The level of Ca^2+^ had significant difference after and before SOST silence (*p < 0.05). Error bar indicates the standard deviation of 3 repeats. (B) MC3T3‐E1 cells were stained with alizarin red S. In the ‘blank contact’ model and the ‘no cell contact’ model, the number of mineralized nodules model had not significant difference between the group with Ti particles and the group without Ti particles. In the ‘cell contact’ model, the number of mineralized nodules significantly decreased in the Ti‐treated group. After SOST inhibition, the number of mineralized nodules increased. The difference in the number of mineralized nodules was significant after and before SOST silence

**FIGURE 8:**
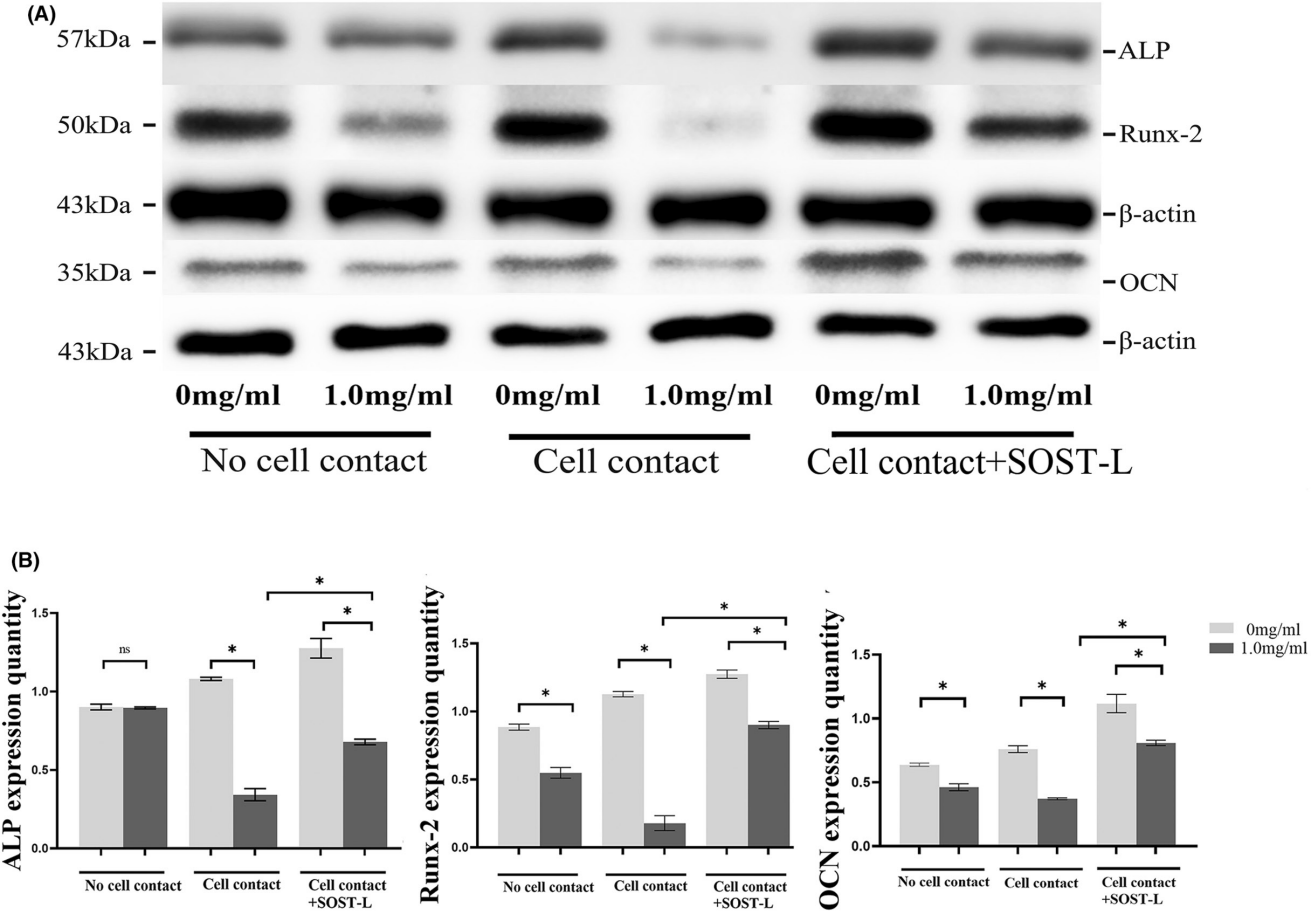
Effects of Ti‐treated MLO‐Y4 cells on the protein expression of ALP, Runx2 and OCN of MC3T3‐E1 cells in the different co‐cultures. (A) In three co‐culture models of ‘no cell contact’, ‘cell contact’ and ‘cell contact with SOST silence’, MLO‐Y4 cells were treated with Ti particles at the concentration of 0 and 1.0 mg/ml and the MC3T3‐E1 cells were collected on day 6 and day 21 separately. The protein expressions of ALP, Runx2 and OCN were detected by western blot and normalized to β‐actin. (B) Data showing in the ‘no cell contact’ model the protein expression of ALP had no significant differences between the group with Ti particles and the group without Ti particles. But the protein expression of Runx2 and OCN had significant differences between the group with Ti particles and the group without Ti particles in the ‘no cell contact’ model. In the ‘cell contact’ model, protein expression of ALP, Runx‐2, and OCN levels decreased significantly in the group with Ti particles compared with the group without Ti particles. After SOST silencing, the levels of ALP, Runx‐2 and OCN went up. The difference in ALP, Runx‐2 and OCN were significant after and before SOST silence in the ‘cell contact’ model (*p < 0.05)

In the ‘no cell contact’ co‐culture, ALP activity in the supernatant and ALP staining had no significant difference in two groups with/without Ti particles (*p* > 0.05) and the protein expression of ALP also had no significant difference in two groups (*p* > 0.05). Similarly, the number of mineralized nodules and Ca^2+^ levels did not differ significantly between the group with Ti Particles and the group without Ti particles in the ‘no cell contact’ co‐culture. While Ti particles decreased the protein expression of Runx2 and OCN in MC3T3‐E1 cells (*p* < 0.05) (Figures 6, 7, 8).

In the ‘cell contact’ model, Ti particles decreased significantly ALP activity of MC3T3‐E1 cells (*p* < 0.05). ALP staining showed fewer positive cells (cytosol blue coloration) in the group with Ti particles compared with the group free from Ti particles. The number of mineralized nodules and Ca^2+^ levels observed in MC3T3‐E1 cells in Ti‐treated group decreased significantly (*p* < 0.05). In addition, the protein expression of ALP, Runx2 and OCN of MC3T3‐E1 cells in the group with Ti particles decreased significantly compared with the group free from Ti particles (*p* < 0.05) (Figures 6, 7, 8).

### 3.5 SOST silence attenuated the inhibition effects of Ti‐induced MLO‐Y4 on osteoblastic differentiation of MC3T3‐E1

After SOST silencing, osteoblastic markers of MC3T3‐E1 cells, such as ALP, Runx2, OCN and mineralized nodules, increased surprisingly even if MLO‐Y4 cells were treated with Ti particles in the ‘cell contact’ co‐culture model. The level of ALP, Runx2, OCN and Ca^2+^ had significant difference after and before SOST silence (*p* < 0.05) (Figures 6, 7, 8).

### 3.6 Ti particles increased Cx43 expression in MLO‐Y4 cells, SOST silence decreased the expression of Cx43 and alleviated the increase of Cx43 in MLO‐Y4 cells induced by Ti particles

In direct cell contact model, MLO‐Y4 cells exposed to Ti particles obviously inhibited MC3T3‐E1 osteoblastic differentiation. We speculated there might exist direct intercellular communication between MLO‐Y4 cells and MC3T3‐E1. Therefore, we examined the expression of Cx43, the major gap junction protein, in MLO‐Y4 cells, and found that the expression of Cx43 increased with the treatment of Ti particle at 24 and 48 h. At the same time, the expression of Cx43 increased significantly with the increasing of Ti particles concentration (*p* < 0.05) (Figure 2B). Through immunofluorescent staining, we verified again Ti particles increased Cx43 expression. Cx43 increased as SOST overexpressed. When SOST silenced, Cx43 expression decreased significantly compared with the control group. Compared with Ti‐treated group, Cx43 expression decreased significantly with the existence of Ti particle after SOST silence (Figure S3).

## 4 DISCUSSION

To understand the mechanism of aseptic loosening, many studies have investigated expression of pro‐inflammatory mediators induced by wear particles^24^, ^25^ and the biological effects of cells around prosthesis such as fibroblasts, monocytes, osteoblasts and osteoclasts.^7^, ^26^, ^27^ However, little study has focused on osteocytes compared with other cell types mentioned above. Our previous in vivo study demonstrated osteocytes played an important role in osteolysis and reduction of SOST promoted bone formation.^22^ In this study, we investigated the effects of Ti particles on MLO‐Y4 osteocytic cells and the effects of Ti‐induced osteocytic alterations on MC3T3‐E1 osteoblastic differentiation in vitro.

Osteocytes possess a specialized cellular morphology and are a stellate shape with numerous dendritic processes. Osteocytes can release several soluble molecules including sclerostin, RANKL, OPG and fibroblast growth factor 23 from lacunae through canaliculi to adjacent bone cells.^13^, ^28^ Meanwhile, osteocytes dendritic processes connect directly to form gap junction, which are transmembrane channels that connect the cytoplasm of adjacent cells and allow the cells to exchange directly small molecules including calcium ions and phosphates.^29^, ^30^ In these two ways, osteocytes play multifunctional roles in orchestrating bone remodelling by regulating both osteoblast and osteoclast function.^11^, ^12^, ^13^, ^14^, ^15^, ^17^, ^31^ When periprosthetic osteolysis occurs, osteocytes expose to the abnormal bone surface and contact directly with implants or wear debris.^32^ Theoretically, osteocyte may regulate bone remodel in periprosthetic osteolysis induced by wear debris.

In our study, Ti particles increased SOST/sclerostin expression and decreased β‐catenin expression instead. Ti particles increased significantly the apoptosis of MLO‐Y4 cells. Ti particles also increased the expression of IL‐6, TNF‐α and Cx43. Ti wear particles had negative effects on MLO‐Y4 osteocytic cells and the impact of Ti particles on the cells was extensive. SOST/sclerostin might play an important role on Ti particles damage to osteocytic cells. SOST silence decreased the expression of IL‐6, TNF‐α, Cx43 and decreased the apoptosis of MLO‐Y4 cells induced by Ti particles. SOST silence increased β‐catenin expression and promoted osteoblastic differentiation. The effects of SOST were multifaceted.

SOST is an osteocytic marker and sclerostin is a soluble secretory protein, the product of SOST.^33^ Osteocytes exclusively release sclerostin through lacunar‐canalicular system to interact other cells. SOST/sclerostin is strongly associated with bone formation via inhibition of canonical Wnt signalling. Mechanical loading decreased sclerostin expression of osteocyte in association with increased bone formation, whereas unloading increased sclerostin level and inhibited bone formation.^34^, ^35^ Virdi et al.^36^ reported that treatment of sclerostin antibody improved implant fixation by promoting bone formation, increased bone‐implant contact and improved trabecular bone volume and architecture. To study the role of osteocytic cells and SOST/sclerostin on controlling osteoblast function, we co‐cultured MLO‐Y4 cells with MC3T3‐E1 cells in the different models. As mentioned above, osteocytes interact with adjacent bone cells through the release of cytokines as well as directly through gap junction. In the ‘no cell contact’ model, MLO‐Y4 cells and MC3T3‐E1 cells communicated only through the medium, which simulated intercellular communication through cytokines released by osteocytes. In ‘cell contact’ co‐cultures, MLO‐Y4 cells and MC3T3‐E1 cells were seeded on two sides of the same porous membrane, allowing direct cell‐to‐cell contact and separating two types of cells for detection easily. That means two types of cells can communicate directly via gap junction on dendritic processes extending through the pores. In these co‐culture models, MC3T3‐E1 cells were not allowed to contact Ti particles, which avoided the effect of Ti particles on osteoblastic cells directly. We used some osteoblastic markers, such as ALP, OCN, Runx2 and extracellular mineralization, to evaluate MC3T3‐E1 cells differentiation in two co‐cultured models.^37^, ^38^, ^39^, ^40^

It should be noted that ALP activity and mineralization of MC3T3‐E1 cells in the ‘cell contact’ model differed from those in the ‘no cell contact’ model. In the ‘no cell contact’ model, ALP activity or mineralization of MC3T3‐E1 cells in Ti particle group had no different from the group without Ti particles. However, Ti particles decreased the protein expression of Runx2 and OCN in osteoblastic cells compared to the group without Ti particles in the ‘no cell contact’ model. These results showed the changed osteocytic cells induced by Ti particles had a weak inhibition effect on osteoblastic differentiation in the ‘no cell contact’ model. We found sclerostin, IL‐6 and TNF‐α in the co‐culture medium increased when MLO‐Y4 cells were exposed to Ti particles. These cytokines might affect osteoblastic markers expression of Runx2 and OCN.^11^, ^19^, ^41^ In the ‘cell contact’ model, ALP activity and mineralization of MC3T3‐E1 cells were conspicuously suppressed in the group treated with Ti particles compared with the group free from Ti particles. In addition, Runx2 and OCN expression of MC3T3‐E1 cells in the group with Ti particles decreased significantly. To further verify the effect of SOST in process of intercellular communication between osteocyte and osteoblast, we silenced SOST and detected that ALP activity and mineralization of MC3T3‐E1 cells increased in the ‘cell contact’ model even if MLO‐Y4 cells were treated with Ti particles. The expression of Runx2 and OCN in osteoblastic cells also increased. SOST silence attenuated the inhibition effects of Ti‐induced MLO‐Y4 cells on MC3T3‐E1 osteoblastic differentiation.

Ti‐induced osteocytic cell changes cause osteoblastic differentiation dysfunction mostly through direct cell‐to‐cell contact. The mechanism of communication between osteocytic cell and osteoblastic cell remains unclear. We speculated that gap junction might be involved in intercellular communication. Gap junction locates in two adjacent layers of cell membrane and is composed of six connexins, which form narrow channels that extend between nearby cells and allow communication between cells via the transfer of small molecules and ions.^30^ To form this structure, it requires direct cell‐to‐cell contact. Fujita et al.^23^ reported that mutual enhancement of differentiation of osteocytes and osteoblasts occurred through direct cell–cell contact. Nishikawa et al.^42^ found that MLO‐Y4 cells upregulate terminal differentiation of MC3T3‐E1 cells via gap junctions. Our study found that Cx43 in MLO‐Y4 cells, the major gap junction protein, increased significantly with the increasing of Ti particles concentration. MLO‐Y4 cells injured by Ti particles might increase the number of gap junctions and more intercellular signals might exchange through gap junctions when two types of cells contact directly.

As mentioned above, the inhibitory effects of MLO‐Y4 cells induced by Ti particles on MC3T3‐E1 osteoblastic differentiation in the ‘cell contact’ model was different from that in the ‘no cell contact’ model. In two co‐culture model, Ti particles induced sclerostin level increase. However, there was no significant difference in sclerostin level exposed to the same concentration of Ti particles between ‘cell contact’ model and ‘no cell contact’ model. How did explain that in the ‘cell contact’ co‐culture model, MLO‐Y4 osteocytic cells with low expression of sclerostin attenuated the inhibitory effects of Ti‐induced osteocytic cells on osteoblastic differentiation? We speculated that there were some reasons for these results. Firstly, the results might be associated with the concentration of sclerostin and cell types. Although MLO‐Y4 cells exposed to Ti particles released more sclerostin, increased sclerostin level in medium was not enough to affect MC3T3‐E1 function. Atkins et al.^43^ reported primary osteoblasts added to recombinant human sclerostin exhibited dose‐ and time‐dependent inhibition of in vitro mineralization. Secondly, sclerostin might not play its roles only as soluble molecules released to adjacent cells. Bivi et al.^44^ reported MLO‐Y4 osteocytic cells with knocked‐down Cx43 exhibited higher β‐catenin protein expression and enhanced response to mechanical stimulation. Cx43 is a Wnt/β‐catenin target gene and the major component of gap junctions in bone cells. SOST/sclerostin, which suppresses bone formation via inhibition of canonical Wnt/β‐catenin pathway, might be associated with gap junction and have a potential role on intercellular communication through gap junction. York et al.^45^ found that gap junctional intercellular communication plays an important role in controlling the sclerostin response of MLO‐Y4 cells to mechanical loads. SOST expression was lower in conditional Cx43 deficient mice relative to WT mice.^46^ Our study showed that SOST silence decreased the expression of Cx43 and alleviated the increase of Cx43 in MLO‐Y4 cells induced by Ti particles. We speculated that SOST played its partial role on the regulation of osteocytic cells over osteoblastic cells through Cx43. Even so, the mechanism of SOST/sclerostin playing the role is still unknown and the next step will be to explore the relationship of Cx43 and SOST/sclerostin in the process of gap junction communication between two types of cells.

Overall, our present study demonstrated that osteocytic cells exposed to wear particles can inhibit osteoblastic differentiation mostly through direct cell‐to‐cell contact and SOST might play an important role on the regulation of osteocyte over osteoblast. These results provided insight into the effect of osteocyte in prosthetic aseptic loosening.

We are aware of the limitations of in vitro studies and cannot draw general conclusion from experiments performed in cell line alone. To validate in the physiological setting, primary cell experiments are undoubtedly the most suitable. However, the multi‐step digestion of bone to receive primary osteocytes is very time consuming and there are only a few cells to be obtained. On the other hand, the culture of primary osteocyte cannot meet our experimental requirements, which SOST need to be silenced. In addition to regulating bone formation, osteocytes also take part in controlling bone resorption. The study about the regulation of osteocyte induced by wear debris on osteoclast should be done in the future.

## AUTHOR CONTRIBUTIONS

**Hao Chai:** Data curation (equal); formal analysis (lead); methodology (equal); software (equal); validation (equal); writing – original draft (equal); writing – review and editing (equal). **Zai Hang Zhang:** Data curation (equal); funding acquisition (equal); methodology (equal); writing – original draft (equal). **Jing Yi Fang:** Data curation (equal); investigation (equal); methodology (equal); writing – original draft (equal). **Chang She:** Formal analysis (equal). **Wei Xu:** Conceptualization (lead); data curation (equal); funding acquisition (equal); project administration (lead); resources (lead); supervision (lead); writing – original draft (equal); writing – review and editing (equal). **De Chun Geng:** Writing – review and editing (equal).

## CONFLICT OF INTEREST

The authors declare no conflict of interest.

## Supporting information


Figure S1

Figure S2

Figure S3

## Data Availability

The authors confirm that the data supporting the findings of this study are available within the article.
