# Supplementary figures and images for: Osteocytic cells exposed to titanium particles increase sclerostin expression and inhibit osteoblastic cell differentiation mostly via direct cell‐to‐cell contact
Source: J Cell Mol Med. 2022 Jun 28;26(15):4371–85. doi: 10.1111/jcmm.17460 (PMC9345295; doi:10.1111/jcmm.17460)

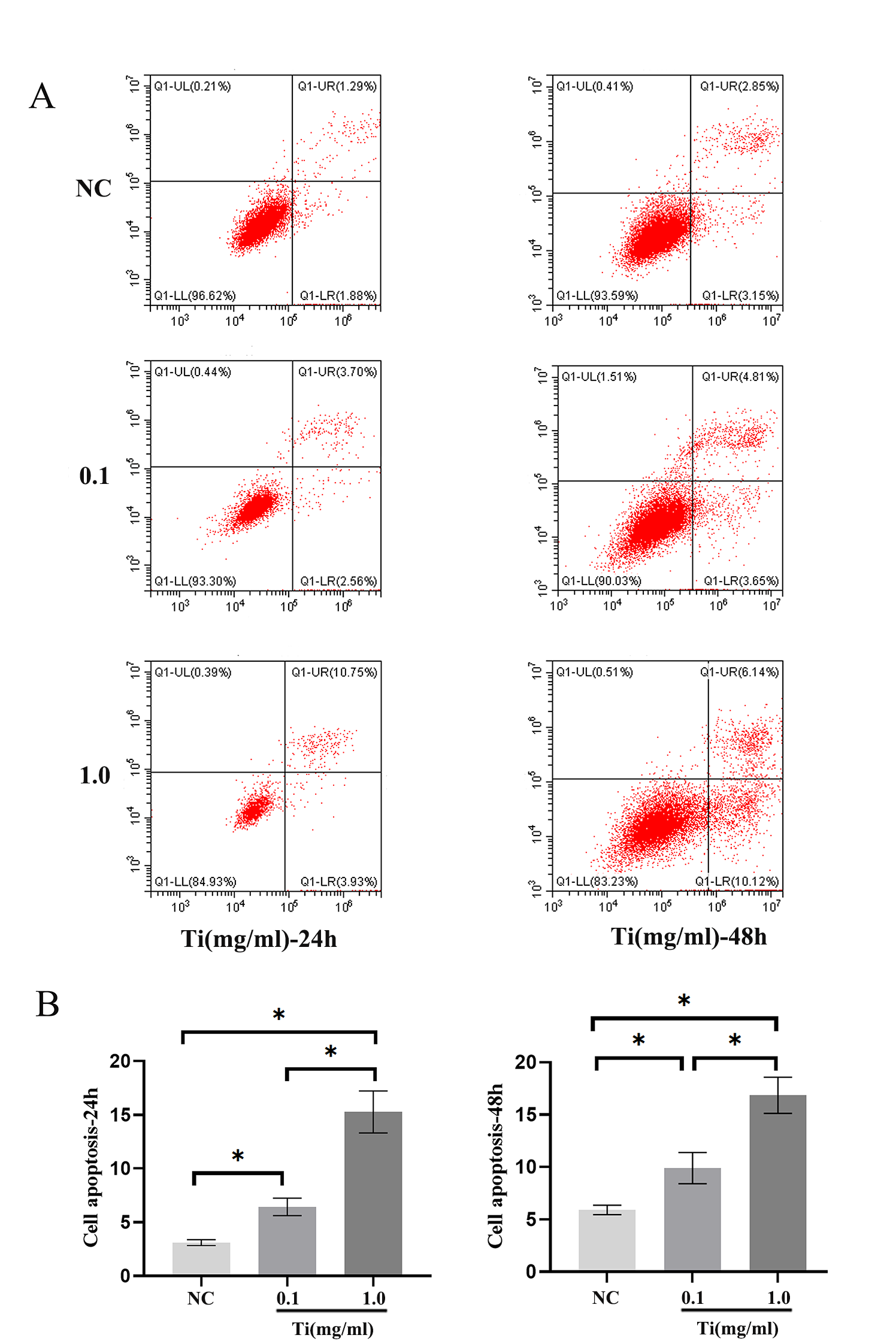

Supplement: Supplementary file 1 — Figure S1 [file JCMM-26-4371-s001.tif]

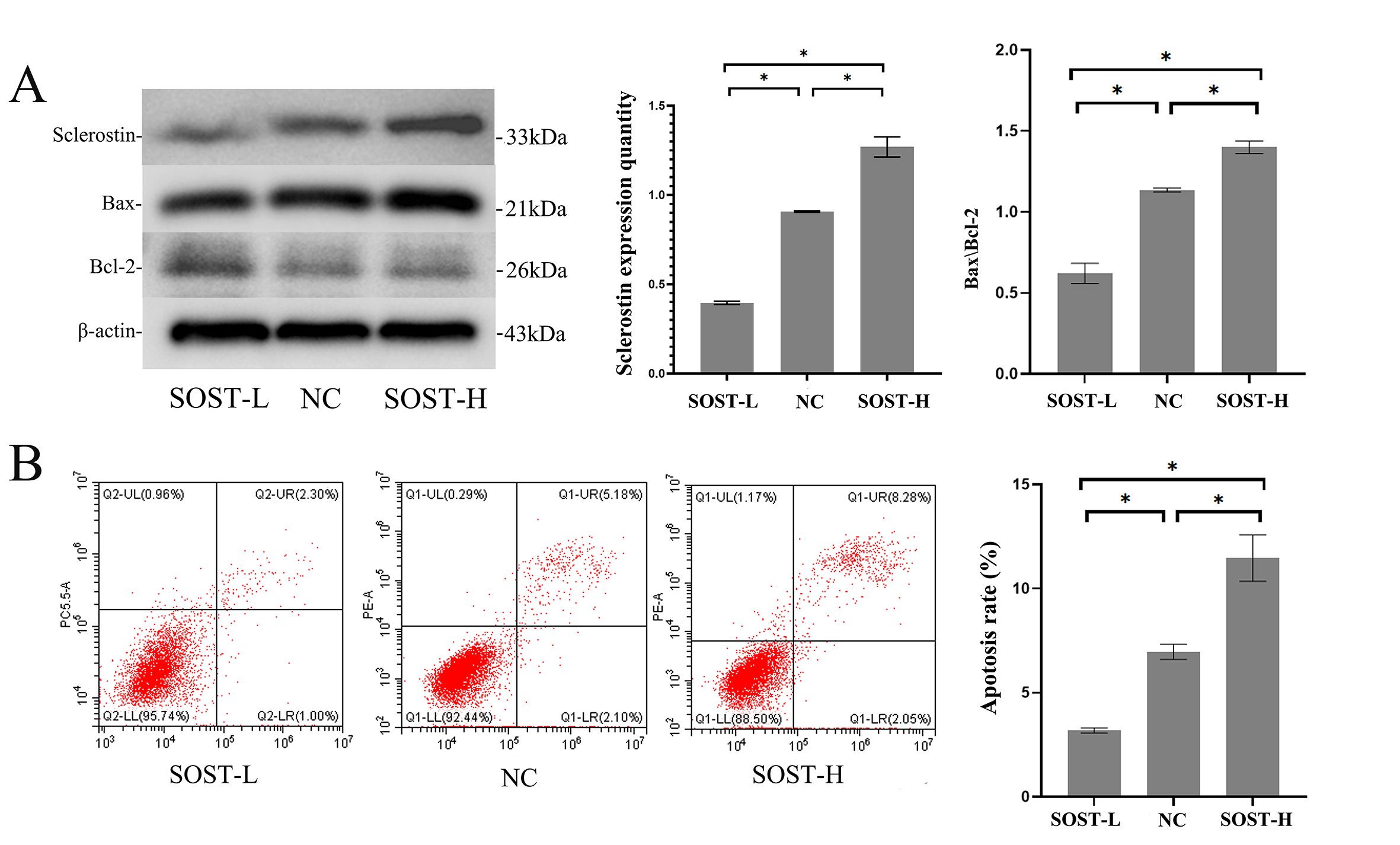

Supplement: Supplementary file 2 — Figure S2 [file JCMM-26-4371-s003.tif]

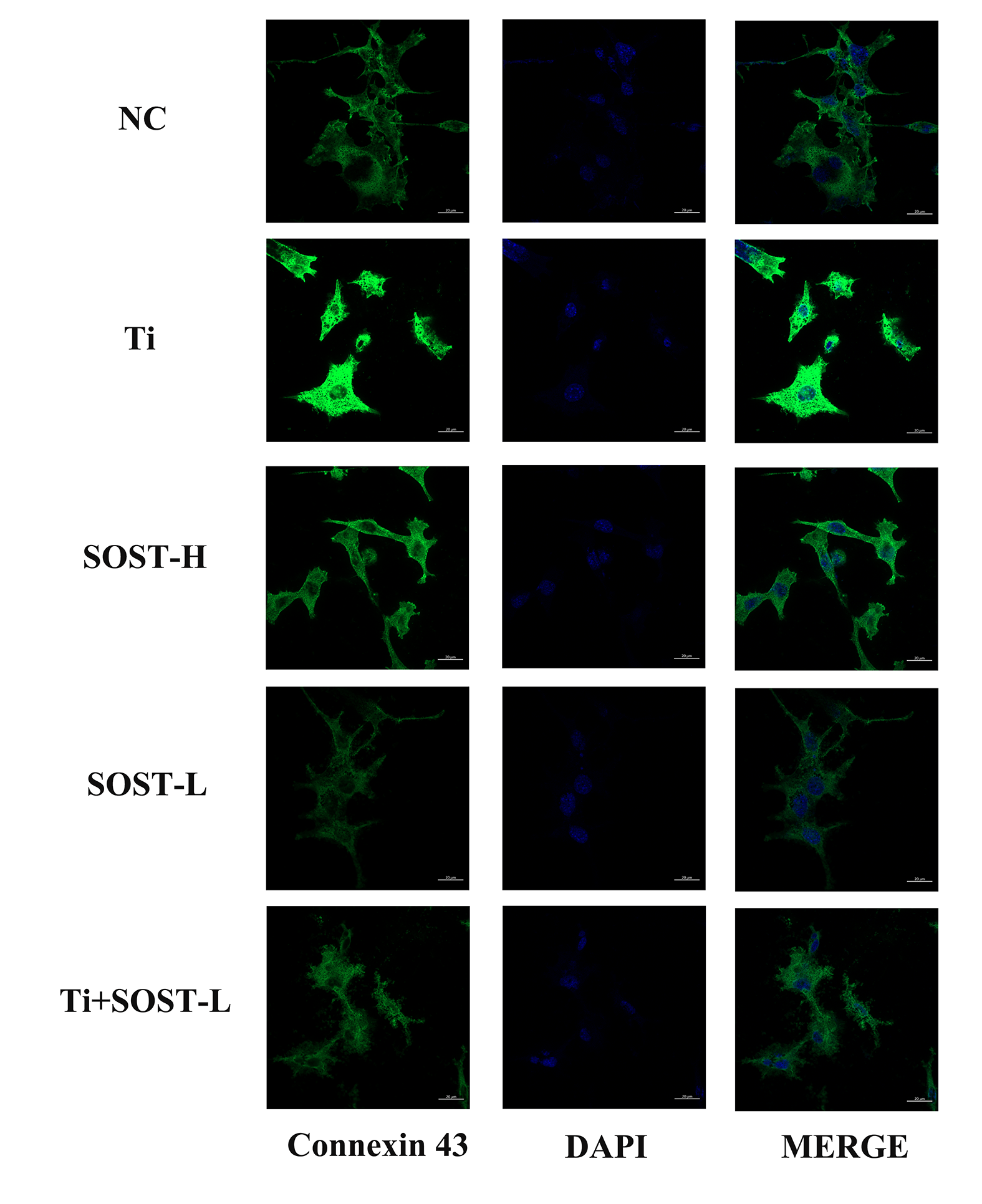

Supplement: Supplementary file 3 — Figure S3 [file JCMM-26-4371-s002.tif]
